# Reported decision-making regarding diuretic use and urinary sodium monitoring in acute heart failure: a vignette-based survey in three European countries

**DOI:** 10.1093/eschf/xvag161

**Published:** 2026-06-04

**Authors:** Niclas Geldermann, Noemi Filidoro, Matthias Paul, Stefan Venturini, Andrea Bürgi, Michael Christ

**Affiliations:** Department of Emergency Medicine, Luzerner Kantonsspital, Kantonsspital 37, Lucerne 6004, Switzerland; University of Lucerne, Faculty of Health Sciences and Medicine, Lucerne, Switzerland; Heart Center, Luzerner Kantonsspital, Lucerne, Switzerland; Department of Emergency Medicine, Luzerner Kantonsspital, Kantonsspital 37, Lucerne 6004, Switzerland; Department of Quality Management, Luzerner Kantonsspital, Lucerne, Switzerland; Department of Emergency Medicine, Luzerner Kantonsspital, Kantonsspital 37, Lucerne 6004, Switzerland

**Keywords:** Heart Failure, Diuretics, Guideline Adherence, Urinary sodium, Diuretic resistance

## Abstract

**Aims:**

To assess clinicians’ decision-making in relation to guideline-recommended diuretic therapy, monitoring, and escalation strategies in acute heart failure (AHF).

**Methods and results:**

We conducted a multinational, case-based cross-sectional survey among clinicians involved in heart failure management in Switzerland, Germany, and Austria. Participants responded to standardized hypothetical clinical vignettes addressing loop diuretic dosing, monitoring, and management of insufficient diuretic response in patients with AHF. Multivariable binomial logistic regression was used to identify factors associated with guideline-concordant initial diuretic dosing and use of urinary sodium measurement. A total of 787 clinicians participated. Guideline-concordant initial loop diuretic dosing was reported by 53.4% of respondents for diuretic-naive patients and by 87.7% for patients receiving chronic diuretic therapy. Treatment monitoring using urinary sodium measurement was reported by 16.9%. In multivariable regression analysis, urinary sodium measurement was more frequent in Switzerland (OR 3.36; 95% CI 2.09–5.42) and Austria (OR 2.64; 95% CI 1.12–6.22) compared to Germany. General practitioners/internal medicine physicians (OR 3.52, 95% CI 1.83–6.79), resident physicians (OR 2.60, 95% CI 1.50–4.51), and prehospital emergency physicians (OR 2.08, 95% CI 1.08–4.03) showed higher odds of urinary sodium measurement compared to emergency medicine physicians. In rural settings, odds were lower compared to urban settings (OR 0.50, 95% CI 0.29–0.88). In case of insufficient diuretic response, 64.6% favoured escalation via sequential nephron blockade.

**Conclusion:**

Reported decision-making showed substantial variability regarding diuretic treatment monitoring and escalation strategies. Urinary sodium measurement was infrequently selected and may represent a potential target for future educational efforts.

## Graphical Abstract

**Graphical Abstract:**
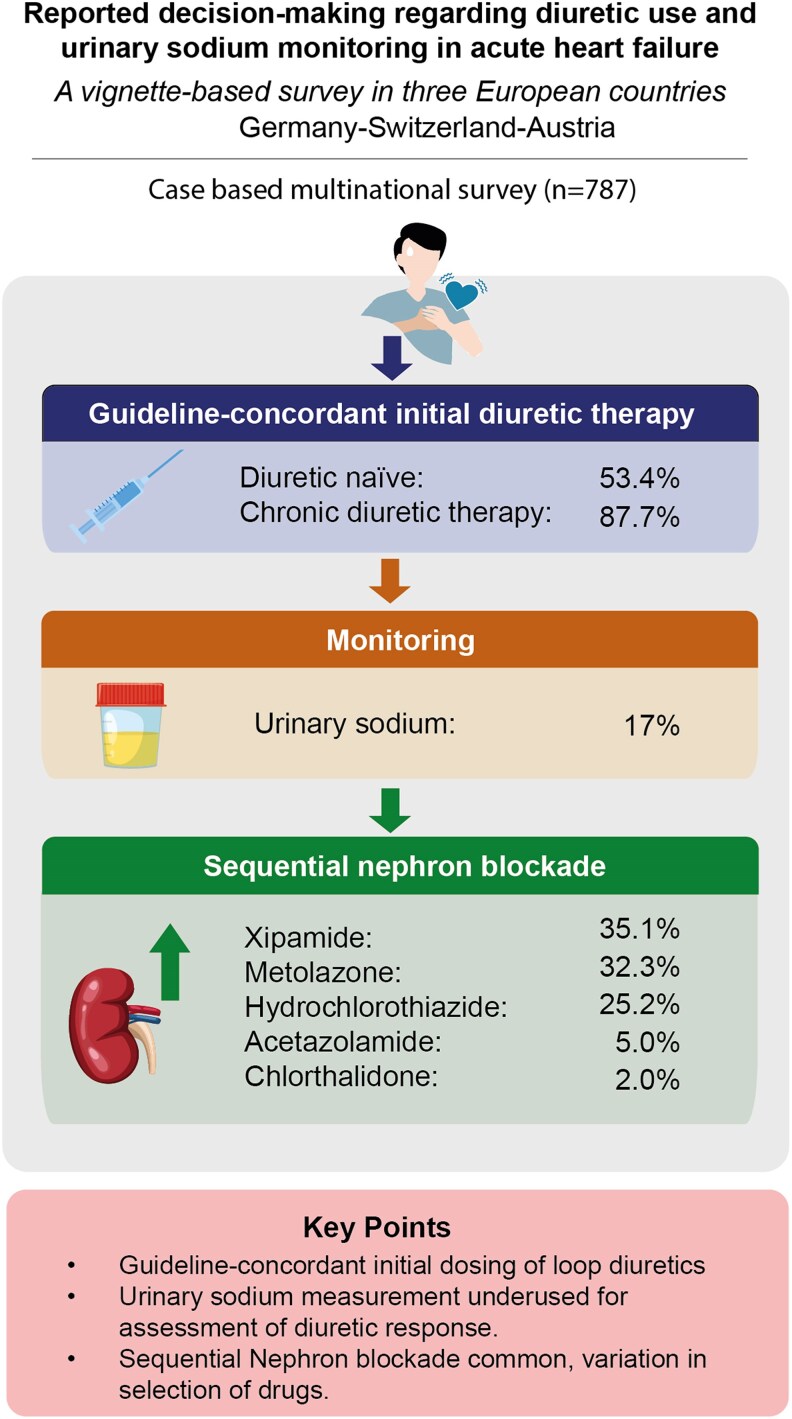

## Introduction

Cardiovascular diseases remain the leading cause of death worldwide, accounting for 42.5% of deaths according to WHO statistics.^1^ The prevalence of heart failure worldwide is high at 1%–2%,^2,3^ and exceeds 10% among those over 70 years of age.^4^ Despite advances in therapy, 5-year mortality after diagnosis of heart failure remains between 53% and 67%.^5^ At a national level, data from the German Federal Health Monitoring System demonstrate a 65% increase in heart failure–related hospitalizations, with heart failure accounting for approximately 45 in-hospital deaths per 10 000 population annually.^6^ This underscores the growing burden of acute heart failure (AHF) on healthcare systems.

Diuretic treatment is the cornerstone in the management of AHF, where congestion remains the leading cause for medical consultation.^7^ In a prospective study of 453 patients with AHF, Oguri et al. showed that greater reduction in congestion scores within the first 3 days was strongly associated with 1-year survival (81.3% vs. 58.3%, *P* < .001).^8^ Yet recent data suggest that decongestion is often insufficiently achieved. In a secondary analysis of a randomized controlled trial on Rolofylline (*n* = 1572), Rubio-Garcia et al. found only 23.5% of patients to be congestion-free at discharge or Day 7, and persistent congestion was associated with increased 60-day rehospitalization rate (hazard ratio (HR) = 1.88; 95% CI = 1.39–2.55) and 180-day mortality (HR = 1.54; 95% CI = 1.16–2.04).^9^

Current European Society of Cardiology (ESC) Guidelines for the diagnosis and treatment of acute and chronic heart failure emphasize standardized treatment with loop diuretics in presenters with AHF and early assessment of diuretic response.^5^ Measurements of urinary output and urinary sodium concentration within the first 6 h are considered essential.^10^ A urinary sodium concentration <50–70 mmol/L identifies insufficient natriuretic response^11^ and is associated with adverse outcomes: in a single-centre, observational, prospective cohort study (*n* = 52), Singh et al. reported a higher risk for the composite endpoint of rehospitalization, transplantation, or death if urinary sodium remained low (Urinary-sodium:Urinary-furosemide ratio <2 mmol/mg) after initial furosemide administration (HR 1.63; 95% CI = 1.10–2.51).^12^ Similar findings were observed in a *post hoc* analysis by Cobo Marcos et al. including 167 patients with ambulatory worsening heart failure, in which lower urinary sodium excretion 3 h after furosemide administration was significantly associated with death, heart failure hospitalization, or need for outpatient intravenous diuretic therapy (OR = 1.29; 95% CI: 1.10–1.51 per decrease in 10 mmol/L urinary sodium).^13^

If the urinary sodium threshold of 70 mmol/L is not reached, evidence supports doubling the diuretic dose and reassessing after 2 h.^5,10^

A substantial proportion of patients with AHF are initially managed by non-cardiologists, particularly in emergency departments (ED) and primary care settings. Only limited evidence is available on how acute care physicians apply guideline recommendations such as urinary sodium measurement after diuretic treatment or approach diuretic resistance in practice. This survey aims to evaluate knowledge of guideline recommendations and management strategies of diuretic resistance among acute care physicians compared with other specialties.

## Methods

### Objectives/aims

To explore current treatment and monitoring practices for diuretic therapy in AHF:

1. Initial dosing strategies in diuretic-naive patients and patients on chronic diuretic therapy.
2. Monitoring of treatment response, especially with urinary sodium measurement.
3. Therapeutic adjustments in case of insufficient response, including dose escalation and sequential nephron blockade.

### Study design

#### Construction and validation of the survey

We conducted a prospective, descriptive, cross-sectional survey. The target population comprised health care providers directly involved in the initial management of patients with AHF, including general practitioners, emergency physicians (prehospital, hospital settings or urgent care units), and relevant specialists such as cardiology or internal medicine. The survey was conducted in German-speaking countries (Switzerland, Austria, Germany).

The questionnaire was developed in accordance with validated methodological recommendations for vignette-based studies of clinicians’ decision-making proposed by Evans et al.^14^ Following initial development, the survey underwent expert review by physicians with expertise in heart failure management. Pilot testing was performed within the ED of Lucerne Cantonal Hospital to assess clarity, comprehensibility, and survey flow prior to deployment.

The survey was initially developed in German and translated into English, Italian, and French using ChatGPT-assisted (OpenAI, San Francisco, California, USA) translation, which showed sufficient translation results for different medical contexts in recent studies.^15,16^ To ensure clarity and linguistic consistency, all translated versions were additionally reviewed by study team members with native language knowledge with particular attention to conceptual equivalence and appropriate medical terminology.

Demographic characteristics of participants were collected. Participants were presented with hypothetical clinical vignettes representing typical AHF presentations. Based on the vignette provided, participants were asked to indicate their preferred diagnostic and therapeutic strategies. Response options were primarily multiple choice with the possibility of additional free-text input. The full set of vignettes is provided in the supplement (Electronic Supplementary S1).

#### Distribution

The survey was distributed using EvaSys platform (EvaSys GmbH, Lüneburg, Germany). Distribution was carried out via social media, mailing lists, and online platforms of relevant professional societies. Reminder emails were sent after 5 and 14 days. Participants had the opportunity to choose between German, English, Italian, or French language. Participation was voluntary and anonymous.

#### Ethics

As the survey relied on fictitious case vignettes and did not involve patient-level data, formal ethics approval was not required according to local regulations. All participants provided informed consent prior to participation.

### Data analysis

Survey data were exported into IBM SPSS statistics (IBM Corp., Armonk, New York, USA) for statistical analysis. As the survey was distributed through multiple open channels, the exact number of clinicians reached cannot be determined. Consequently, no formal response rate can be calculated. Descriptive statistics were used to summarize participant characteristics and response patterns regarding diuretic dosing, monitoring strategies, and therapeutic adjustments in the case of insufficient diuretic response. Categorical variables are presented as absolute numbers and percentages. Partially completed questionnaires were included in the analysis using available responses on a per-question basis. Multivariable binomial logistic regression was performed with Jamovi (Jamovi, Sydney, Australia) to identify factors associated with the use of urine sodium measurement and the selection of guideline-concordant initial loop diuretic dosing strategies in patients receiving chronic diuretic therapy. A forest plot was created using Excel (Microsoft Corporation, Redmond, WA, USA). All independent variables were included as categorical variables. Reference categories were defined as the most frequent or clinically standard category to ensure interpretability of odds ratios. Because several original age categories contained only small numbers of participants, age was collapsed into broader categories (<35, 35–54, and ≥55 years) for multivariable analysis to improve robustness and stability of model estimates. In multivariable regression, only participants with complete data for all variables included in the respective model were analysed to provide stable estimation within regression models.

## Results

### Study population

A total of 787 physicians from Switzerland, Germany, and Austria participated in the survey from August until October 2025. Three participants were excluded due to missing consent for data processing, leaving 784 participants for final analysis. Response completeness remained high throughout the first three clinical vignettes (*n* = 783), while incomplete responses were observed mainly in subsequent subquestions of later survey sections.

Most respondents were based in Germany (*N* = 377; 48.1%) and Switzerland (*N* = 358; 45.7%). 6.1% (*N* = 48) of respondents practiced in Austria. Nearly half of the participants worked in ED (*N* = 383; 49.0%), followed by prehospital physicians (*N* = 114; 14.6%). General practitioners or internal medicine physicians accounted for 9.7% (*N* = 76) of respondents and 1.7% (*N* = 13) worked in ambulatory urgent care. 16.3% (*N* = 127) of participants were resident physicians. The majority of physicians practiced in urban regions (*N* = 472; 60.4%). Most respondents were aged between 35 and 54 years, and 33.5% (*N* = 261) were female. Baseline characteristics of participants are summarized in *Table 1*.

**Table 1:** Baseline characteristics of physicians participating in the survey

Characteristic		*N* (%)
Total		784 (100)
Country	Switzerland	358 (45.7)
	Germany	377 (48.1)
	Austria	48 (6.1)
Specialty	General practitioner/internal medicine physician	76 (9.7)
	Ambulatory urgent care unit	13 (1.7)
	Emergency department	383 (49)
	Prehospital emergency physician	114 (14.6)
	Resident physician	127 (16.3)
	Other	68 (8.7)
Region	Urban (e.g. metropolitan area or major city)	472 (60.4)
	Peri-urban (suburban or urban fringe areas)	106 (13.6)
	Rural (small towns or rural regions)	203 (26)
Age group (years)	18–24	1 (0.1)
	25–34	182 (23.4)
	35–44	289 (37.1)
	45–54	193 (24.8)
	55–65	93 (11.9)
	>65	18 (2.3)
	Not reported	3 (0.4)
Sex	Female	261 (33.5)
	Male	495 (63.5)
	Not reported	24 (3.1)

### Initial loop diuretic dosing strategies

**Figure 1:**
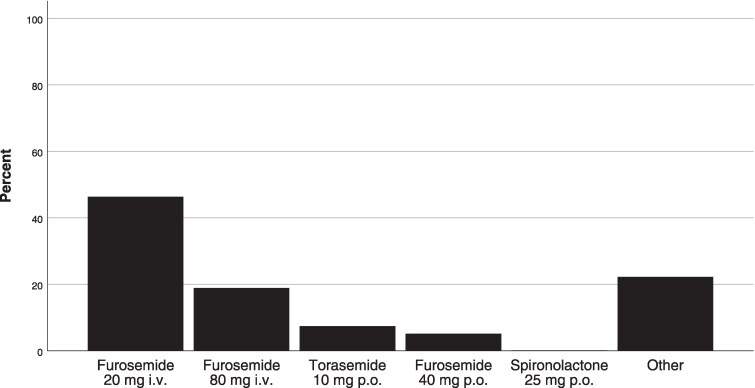
Initial diuretic choice in a clinical scenario of acute heart failure in a diuretic-naive patient. Distribution of selected initial diuretic regimens among survey participants: furosemide 20 mg i.v. (*n* = 363), furosemide 80 mg i.v. (*n* = 148), torasemide 10 mg p.o. (*n* = 58), furosemide 40 mg p.o. (*n* = 40), spironolactone 25 mg p.o. (*n* = 1), and other treatment strategies (*n* = 174). Bars represent the proportion of respondents selecting each option.

Case 1 described a diuretic-naive patient presenting signs and symptoms of acute decompensated heart failure. In diuretic-naive patients, intravenous (i.v.) loop diuretics were the recommended initial treatment strategy. Low-dose i.v. furosemide (i.e. 20 mg furosemide i.v.) was selected most frequently, followed by higher i.v. doses, whereas oral loop diuretics and other agents were chosen less often (*Figure 1*). When analyzing free-text answers for the option ‘other’, 56 (7.1%) participants chose to give 40 mg of furosemide i.v.

**Figure 2:**
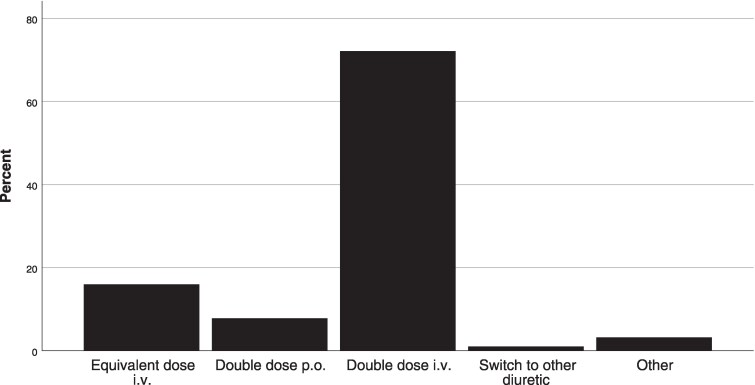
Treatment strategies in a patient on chronic diuretic therapy with AHF. Administration of an equivalent dose i.v. (*n* = 125), doubling of the oral dose (*n* = 61), doubling of the i.v. dose (*n* = 565), switch to another diuretic (*n* = 8), and other strategies (*n* = 25). Bars represent the proportion of respondents selecting each option.

In patients receiving chronic oral loop diuretic therapy (Case 2: 10 mg torasemide per day), most respondents favoured i.v. dose escalation during acute decompensation. Most participants selected doubling of the equivalent loop diuretic dose administered i.v., while continuation of the equivalent of the home dose or switching to an alternative diuretic was chosen less frequently (*Figure 2*).

In multivariable logistic regression (*N* = 771) assessing factors associated with guideline-concordant initial loop diuretic dosing (same or double equivalent dose loop diuretics i.v.) in patients receiving chronic diuretic therapy, physicians aged <35 years were more likely to select a guideline-concordant dosing strategy compared with participants aged 35 to 54 years (OR 2.65, 95% CI 1.18–5.95, *P* = .02). No significant associations were observed for country, specialty, practice region, or sex (Electronic Supplementary S2).

### Monitoring of treatment response after initial diuretic therapy

Early reassessment after initiation of diuretic therapy was widely endorsed, with 94% (*N* = 733) of respondents indicating that treatment response should be re-evaluated within the first hours, but measurement of urinary sodium concentration was reported by only 16.9% (*N* = 121) of all participants. (*Table 2*).

**Table 2:** Monitoring of treatment response after initial diuretic therapy

Strategy	*N* (%)
Planned reassessment	733 (94.2)
Timing of reassessment	
30 min	47 (6.4)
After 1 h	163 (22.3)
After 2 h	264 (36.1)
After 4 h	147 (20.1)
In 2–3 days	108 (14.8)
Other	2 (0.3)
Urinary sodium measurement	121 (16.9)

**Figure 3:**
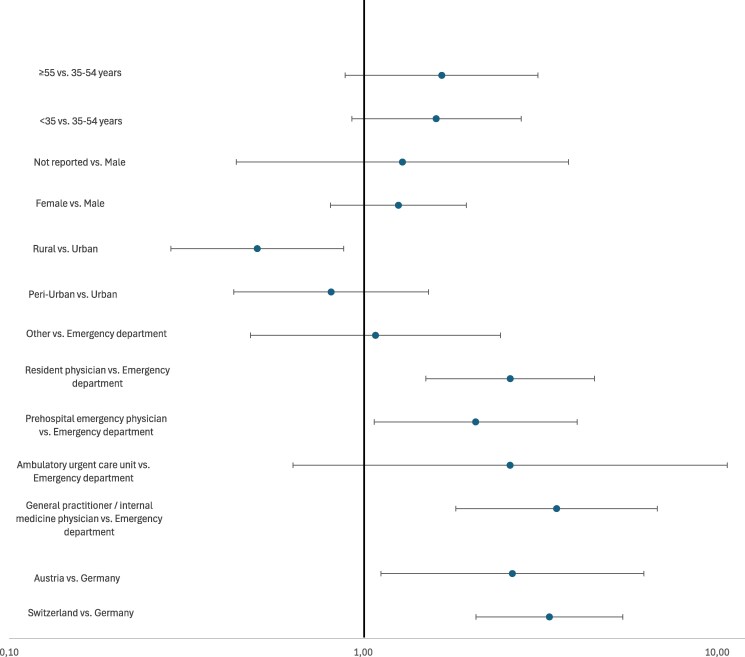
Multivariable binomial logistic regression of factors associated with the use of urinary sodium measurement. Forest plot displays odds ratios (OR) with 95% confidence intervals (CI) for the association between physician characteristics and the reported use of urinary sodium measurement for reassessment of diuretic therapy. For each variable, the category with the highest number of responses was used as the reference group, which is placed on the right. The vertical reference line indicates an OR of 1.0.

In multivariable binomial logistic regression, data of 703 patients were analysed. The use of urinary sodium measurement was significantly associated with country and professional background. Compared with Germany, urinary sodium measurement was more frequently reported in Switzerland and Austria. General practitioners/internal medicine physicians, resident physicians, and prehospital emergency physicians showed higher odds of urinary sodium measurement compared with ED physicians. Practice in rural settings was associated with lower odds of urinary sodium measurement compared with urban settings, whereas sex of physicians was not significantly associated with urinary sodium measurement (*Table 3*, *Figure 3*).

**Table 3:** Factors associated with the use of urinary sodium measurement in patients with AHF, assessed by binomial logistic regression

Variable	Odds ratio	95% CI	*P* value
Country			
Switzerland vs. Germany	3.36	2.09–5.42	<.001
Austria vs. Germany	2.64	1.12–6.22	.026
Specialty			
General practitioner/Internal medicine vs. emergency department	3.52	1.83–6.79	<.001
Ambulatory urgent care unit vs. emergency department	2.60	0.63–10.69	.184
Prehospital emergency physician vs. Emergency department	2.08	1.08–4.03	.029
Resident physician vs. emergency department	2.60	1.50–4.51	<.001
Other vs. emergency department	1.08	0.48–2.45	.844
Region			
Peri-urban vs. urban	0.81	0.43–1.53	.521
Rural vs. urban	0.50	0.29–0.88	.016
Sex			
Female vs. male	1.26	0.81–1.96	.307
Not reported vs. Male	1.29	0.44–3.81	.642
Age			
<35 vs. 35–54 years	1.61	0.93–2.8	.09
≥55 vs. 35–54 years	1.67	0.89–3.12	.11

Analysis was performed using sample size *N* = 703.

### Therapeutic adjustments in case of insufficient response to initial diuretic regimen

In the third case scenario, participants were presented with a patient showing insufficient diuretic response, defined as inadequate urine output despite treatment with i.v. furosemide at a total daily dose of 120 mg.

**Figure 4:**
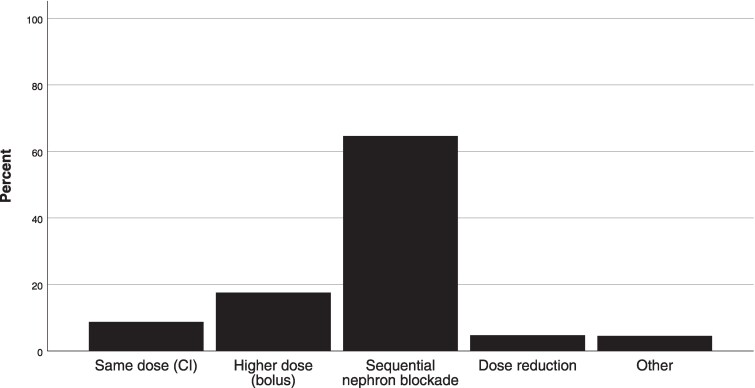
Management strategies in case of insufficient response to diuretics (diuretic resistance). Reported therapeutic approaches in the setting of inadequate diuretic response, shown from left to right: continuation of the same dose as continuous infusion (CI) (*n* = 68), administration of a higher dose as bolus (*n* = 137), sequential nephron blockade (*n* = 506), dose reduction due to concerns about renal function (*n* = 37), and other strategies (*n* = 35). Bars represent the proportion of respondents selecting each option.

Most respondents suggested escalating diuretic therapy by initiating sequential nephron blockade (*N* = 506/783; 64.6%), primarily through the addition of a thiazide or thiazide-like diuretic, or a carbonic anhydrase inhibitor. 16.5% (*N* = 137/783) selected further escalation of loop diuretic dosing via bolus administration (*Figure 4*).

Initiation of combination diuretic therapy as sequential nephron blockade was most considered appropriate after 12–24 h of persistently insufficient urine output despite high-dose loop diuretic therapy (*N* = 336/505; 66.5%).

When asked to specify the preferred add-on agent, substantial heterogeneity was observed. The most frequently selected drugs were xipamide (*N* = 177/504; 35.1%), metolazone (*N* = 163/504; 32.3%), and hydrochlorothiazide (*N* = 127/504; 25.2%), whereas acetazolamide (*N* = 25/504; 5.0%) and chlorthalidone (*N* = 10/504; 2.0%) were chosen less often.

## Discussion

In this multinational, case-based survey across three European countries, we evaluated current knowledge regarding initial diuretic therapy and monitoring in patients with AHF. By focusing on case-based clinical decision-making across different healthcare settings, this study provides insight into clinicians’ reported approaches to guideline-recommended care beyond initial prescribing, an area that remains poorly characterized in contemporary heart failure research. Several important findings emerged.

### Initial dosing of loop diuretics

According to the 2021 ESC Guidelines for the diagnosis and treatment of acute and chronic heart failure, an initial i.v. dose of 20–40 mg furosemide is recommended for diuretic-naive patients with AHF.^5^ 46.3% of participants selected the guideline-recommended initial dose of 20 mg intravenous furosemide. In the free-text responses, 7.1% of participants indicated that they would administer 40 mg i.v. Even when these responses were considered appropriate, only 53.4% of respondents selected a correct initial loop diuretic dose. However, omission of 40 mg i.v. furosemide as a predefined response option may have introduced instrument bias and could have resulted in underestimation of guideline-concordant responses in this vignette. Therefore, these findings should be interpreted cautiously. Nevertheless, the observed variability in responses suggests heterogeneity in reported decision-making regarding initial loop diuretic dosing in AHF.

ESC Guidelines recommend an initial i.v. furosemide dose equivalent to 1–2 times the patient’s usual daily oral dose in patients receiving chronic diuretic treatment. Considering options ‘same dose i.v.’ and ‘double dose’ as appropriate, 87.7% of participants selected a guideline-concordant dosing strategy. This suggests a high level of adherence to recommendations for this case scenario. Interestingly, participants aged <35 years showed a significantly higher likelihood of selecting a guideline-concordant initial dosing strategy compared with the 35–54-year age group. The reasons for this finding remain uncertain, particularly as no significant associations were observed for specialty or practice setting. Therefore, these exploratory findings should be interpreted cautiously.

It should be noted that recommendations in the 2022 ACC/AHA/HFSA Guideline for the Management of Heart Failure differ slightly from the ESC Guidelines, suggesting a higher initial furosemide dose of 40–80 mg in diuretic-naive patients and an initial dose of 1.5–2 times the chronic daily diuretic dose in patients receiving long-term diuretic therapy.^17^ This discrepancy among international guidelines may have influenced the variation in dosing choices observed among our participants.

The clinical relevance of appropriate initial loop diuretic dosing is supported by several studies in current literature: In a *post hoc* analysis of a prospective multicentre study including 1093 patients with AHF, Yoshioka et al. compared length of hospital stay and 60-day mortality across three dosing groups (lower than recommended, recommended, and higher than recommended). Patients receiving lower-than-recommended doses had a significant longer hospitalization (median increase of 3 days), whereas higher-than-recommended doses were associated with increased 60-day mortality (HR = 2.05; 95% CI 1.09–3.88). These findings support the importance of correct initial dosing and reinforce the clinical implications of possible practice gaps identified in our study.^18^

### Use of urinary sodium measurement

Reported use of urinary sodium measurement for monitoring diuretic response showed substantial variability among participants. Although early reassessment of treatment response was widely endorsed, objective monitoring using urinary sodium concentration was selected by only 16.9% of respondents. Given the potential overrepresentation of clinicians with a particular interest in heart failure management, actual implementation in broader routine clinical practice may be even lower.

These findings contrast with accumulating evidence highlighting the clinical relevance of urinary sodium as an early and reliable marker of diuretic response in AHF. In a prospective cohort study of 111 patients with AHF, patients with a decrease or no change in urinary sodium within the first 48 h experienced less effective decongestion, as reflected by lower weight loss (2.5 ± 2.0 vs. 4.5 ± 5.0 kg), and a higher rate of incomplete decongestion at discharge (14% vs. 4%; *P* < .05).^19^ Furthermore, 1-year survival was significantly lower in patients with a decrease or no change in urinary sodium at 6 and 48 h compared with those showing an increase (42% vs. 14% and 31% vs. 16%; *P* < .05).^19^

Despite these associations, the benefit of natriuresis-guided therapy on hard clinical outcomes remains uncertain. In a prospective study including 310 patients with AHF, Ter Maaten et al. demonstrated higher cumulative sodium excretion after 24 h in a natriuresis-guided treatment group compared with standard care (409 ± 178 vs. 345 ± 202 mmol; *P* = .0061), without a corresponding reduction in the composite endpoint of all-cause mortality or first heart failure rehospitalization at 180 days.^20^ The ENACT-HF-study, a multicentre prospective study with 401 patients, supported these findings, adding that a natriuresis-guided therapy shortened duration of hospitalization by a mean of 1.2 days (*P* = .036).^21^

In multivariable analysis, country, specialty, and practice setting were associated with reported urinary sodium measurement, whereas age and sex were not. These differences may reflect variability in local practice patterns, workflow structures, familiarity with guideline recommendations, or access to diagnostic resources. Interestingly, urinary sodium measurement was reported less frequently by ED physicians, despite guideline recommendations emphasizing early reassessment during the initial treatment phase. One possible explanation may be the time-sensitive and resource-limited environment of emergency care, where additional diagnostic procedures may be more difficult to integrate into routine workflows.

One potential strategy to address this challenge has been evaluated in the EASY-HF study, a prospective randomized controlled trial comparing a nurse-led, natriuresis-guided protocol with standard care in 60 patients with AHF. Using point-of-care urinary sodium assessment performed by nursing staff, natriuresis after 48 h was significantly higher in the intervention group compared with standard care (820 ± 279 vs. 657 ± 273 mmol; *P* = .027).^22^ This suggests that task delegation and point-of-care diagnostics may help mitigate time constraints in the ED and support the implementation of urinary sodium–guided management strategies.

Further biomarkers were recently evaluated to measure diuretic response. Iwanek et al. evaluated the adjustment of urinary sodium concentration for urine dilution to improve the prediction of poor early diuretic response. In a prospective observational study including 111 patients with AHF, the urinary sodium/creatinine ratio measured 2 h after intravenous diuretic administration outperformed the guideline-recommended urinary sodium cut-off alone (2.5 times higher OR to identify poor diuretic response).^23^ Similarly, recent data suggest that urinary chloride concentration may represent another promising marker of early diuretic response. In a prospective study including 50 patients with AHF, urinary chloride <72 mmol/L after furosemide administration was strongly associated with poor diuretic response (OR 39.0; 95% CI 3.8–405.0) and demonstrated superior prognostic performance compared with urinary sodium alone.^24^

Further prospective studies are needed to better define the role of biomarker-guided strategies and their impact on clinically relevant outcomes in routine practice.

### Reaction to diuretic resistance

When confronted with an insufficient diuretic response, most respondents indicated escalation of therapy by initiating sequential nephron blockade. A relevant proportion opted for further escalation of loop diuretic dosing. Given the limited evidence and lack of standardized guidance regarding optimal timing and escalation strategies, these responses both likely reflect reasonable approaches.^25^ Evidence from recent randomized trials supports improved decongestion with sequential nephron blockade. In the CLOROTIC trial (*n* = 230), the addition of hydrochlorothiazide resulted in 53% greater weight loss after 5 days compared with placebo.^26^ Similarly, in the ADVOR trial, adjunctive acetazolamide in 519 patients significantly increased rates of successful decongestion (42.2% vs. 30.5%; *P* < .001).^27^ Notably, none of the trials demonstrated a reduction in rehospitalization rates or mortality.

Despite this evidence, neither the optimal timing of escalation nor the selection of add-on agents is well defined. In a randomized, double-blind trial of 60 patients with AHF and diuretic resistance, weight loss did not differ significantly between metolazone, chlorothiazide, and tolvaptan as add-on therapies.^28^ Current guidelines therefore provide limited guidance regarding escalation timing and preferred combination regimens, likely contributing to the heterogeneity observed in our study.^5^ Differences in the reported use may additionally reflect regional availability, prescribing habits, and familiarity with specific agents rather than guideline interpretation alone.

The findings underscore the need for further studies to optimize escalation strategies and to support more timely, individualized management of persistent congestion in AHF.

## Limitations

Some limitations of the study should be acknowledged. The cross-sectional survey design does not allow causal inference, and findings are based on self-reported responses to hypothetical clinical scenarios rather than observed clinical practice. In addition, the open distribution strategy via professional societies, mailing lists, and social media prevented calculation of a formal response rate and introduced the possibility of selection bias. The number of survey views or openings could not be determined retrospectively. Clinicians with a particular interest in AHF management or guideline-based care may have been more likely to participate. Consequently, the proportion of guideline-concordant responses observed in this survey may overestimate adherence compared with a broader and less selectively interested clinician population. Potentially relevant factors such as years in practice, hospital type, academic affiliation, or heart-failure-specific training were not assessed and may have influenced reported decision-making. Furthermore, the relatively small proportion of participants from Austria limits the interpretation of country-specific comparisons. While participant numbers remained largely complete throughout the first three clinical vignettes, attrition was observed in later survey sections, potentially related to survey length.

Nevertheless, the present study has several notable strengths. The large sample size, including more than 750 physicians from different specialties, practice settings, and three European countries, provides a broad overview of reported decision-making in AHF. The vignette-based design allowed standardized assessment of responses to identical hypothetical clinical scenarios across different physician groups and may help identify areas for future prospective research and targeted educational initiatives.

## Conclusions

In this cross-sectional study based on hypothetical case scenarios, variability in responses regarding diuretic management of AHF was observed across countries, professional groups, and care settings. While guideline-concordant initial loop diuretic dosing was frequently reported, differences were noted in treatment monitoring, escalation strategies, and the use of urinary sodium measurement. Our findings suggest different factors may influence diuretic decision-making and may help identify areas for future educational initiatives and prospective research.

## Supplementary Material

xvag161_Supplementary_Data

## Declarations

### Data availability

The data underlying this article will be shared on reasonable request to the corresponding author.

### Pre-registered Clinical Trial Number

None supplied.
