# Supplementary material for: Reported decision-making regarding diuretic use and urinary sodium monitoring in acute heart failure: a vignette-based survey in three European countries
Source: ESC Heart Fail. 2026 Jun 4;13(3):xvag161. doi: 10.1093/eschf/xvag161 (PMC13282897; doi:10.1093/eschf/xvag161)
Supplement: xvag161_Supplementary_Data [file xvag161_supplementary_data.zip › Supplement 2.docx]

| Variable | Odds ratio | 95%-CI | p value |
| --- | --- | --- | --- |
| **Country** |  |  |  |
| Switzerland vs. Germany | 1.21 | 0.74–1.94 | 0.44 |
| Austria vs. Germany | 0.69 | 0.28–1.67 | 0.40 |
| **Specialty** |  |  |  |
| General practitioner / Internal medicine vs. Emergency department | 0.55 | 0.28–1.10 | 0.09 |
| Prehospital emergency physician vs. Emergency department | 0.57 | 0.31–1.03 | 0.06 |
| Resident physician vs. Emergency department | 0.94 | 0.39–2.30 | 0.9 |
| Other vs. Emergency department | 1.28 | 0.51–3.24 | 0.6 |
| **Region** |  |  |  |
| Peri-Urban vs. Urban | 0.77 | 0.40–1.49 | 0.44 |
| Rural vs. Urban | 0.76 | 0.45–1.28 | 0.3 |
| **Sex** |  |  |  |
| Female vs. Male | 0.94 | 0.56–1.56 | 0.8 |
| **Age** |  |  |  |
| <35 vs. 35-54 years | 2.65 | 1.18–5.95 | 0.02 |
| ≥55 vs. 35-54 years | 0.65 | 0.37–1.14 | 0.13 |

Supplement 2: Multivariable logistic regression analysis assessing factors associated with guideline-concordant initial loop diuretic dosing in patients receiving chronic diuretic therapy. Younger physicians (<35 years) were significantly more likely to select a guideline-concordant dosing strategy compared with participants aged 35–54 years, while no significant associations were observed for country, specialty, practice region, or sex. Analysis was performed using sample size N=771.
