# Supplementary figures and images for: Reported decision-making regarding diuretic use and urinary sodium monitoring in acute heart failure: a vignette-based survey in three European countries
Source: ESC Heart Fail. 2026 Jun 4;13(3):xvag161. doi: 10.1093/eschf/xvag161 (PMC13282897; doi:10.1093/eschf/xvag161)

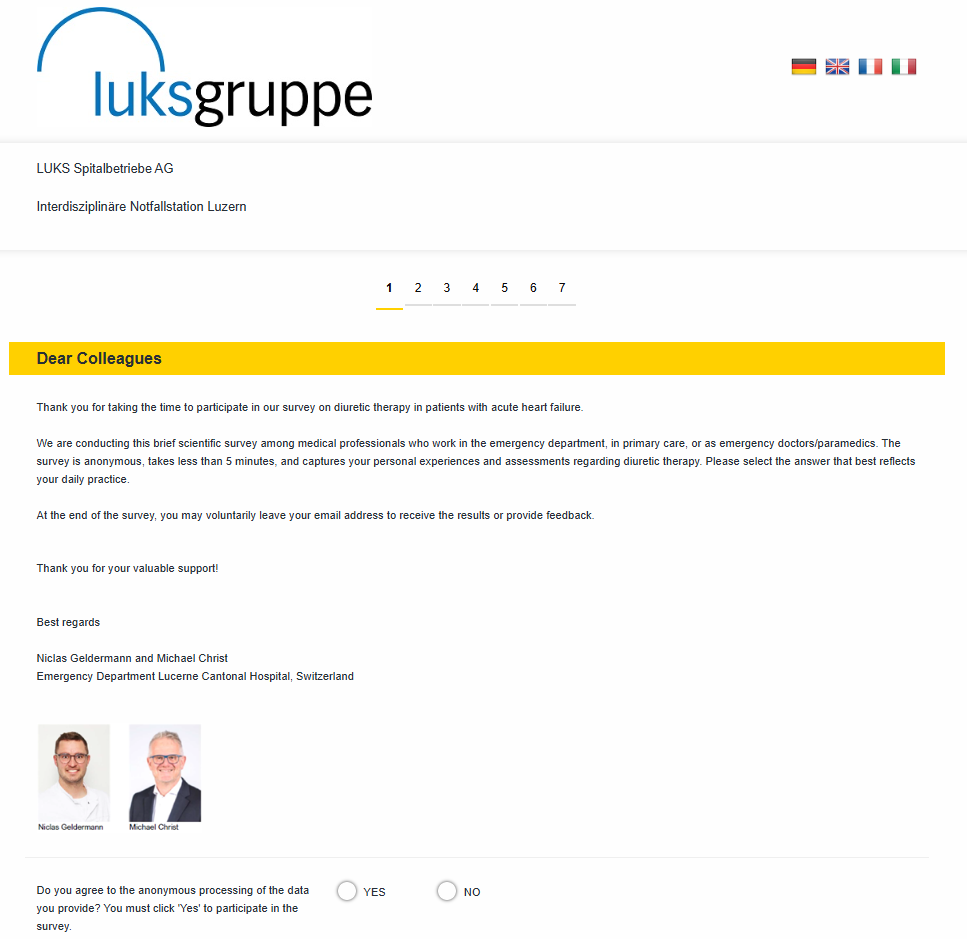


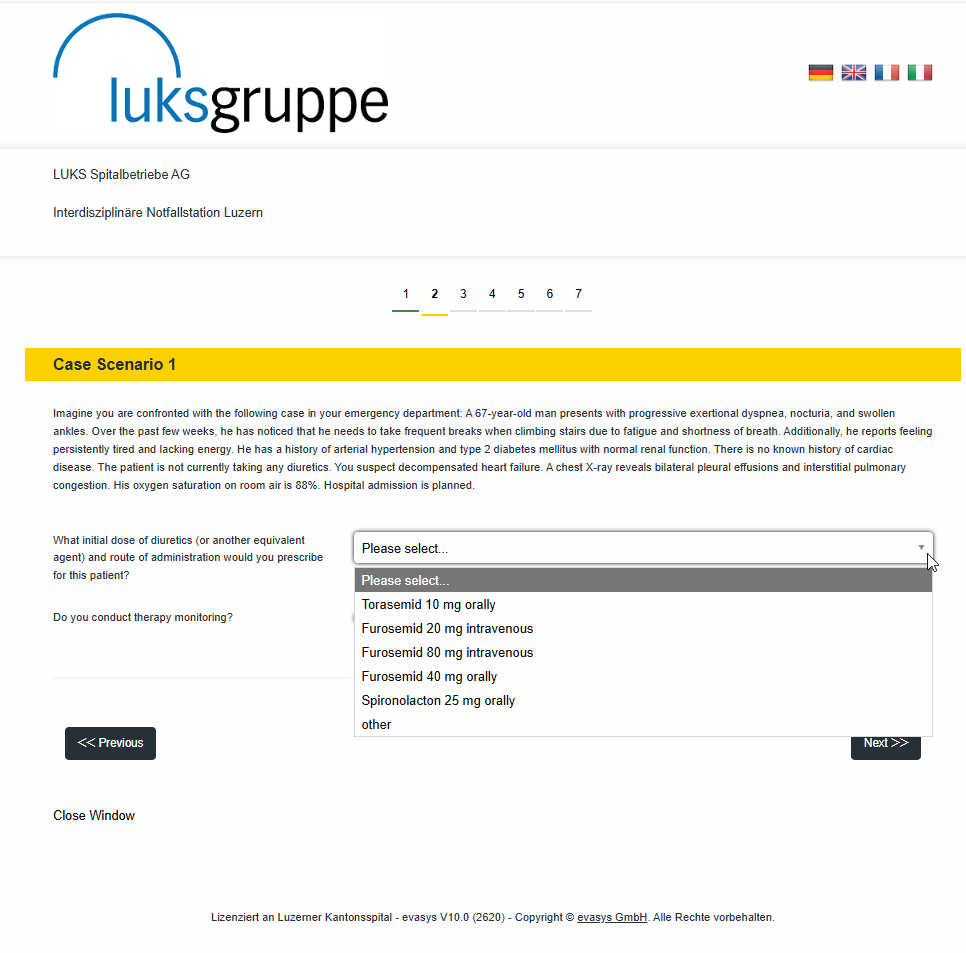


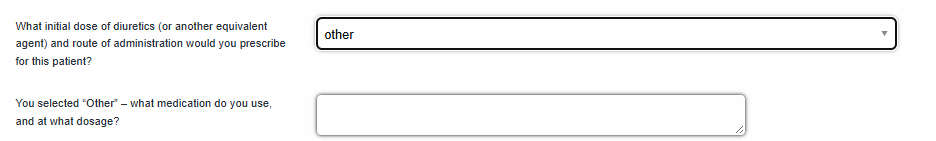


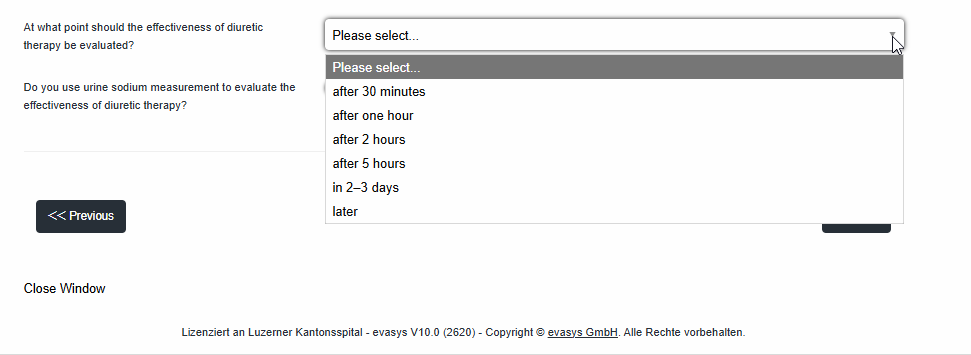


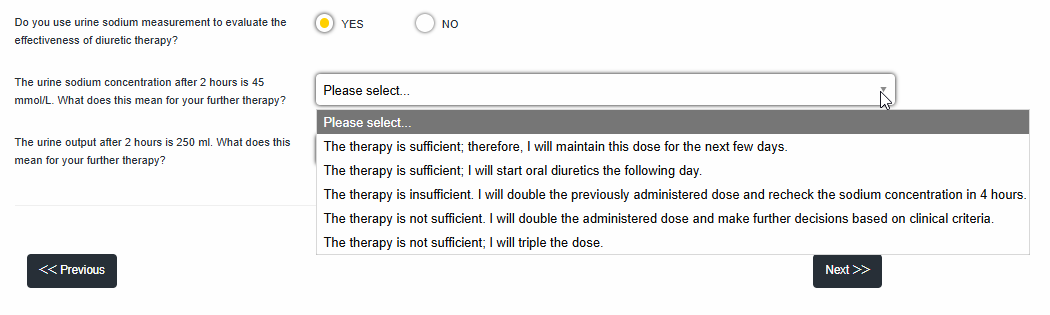


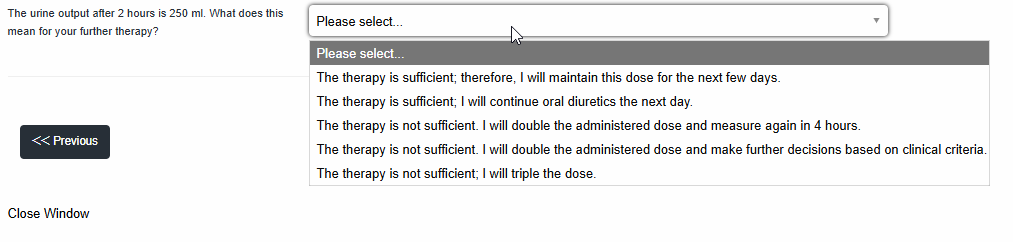


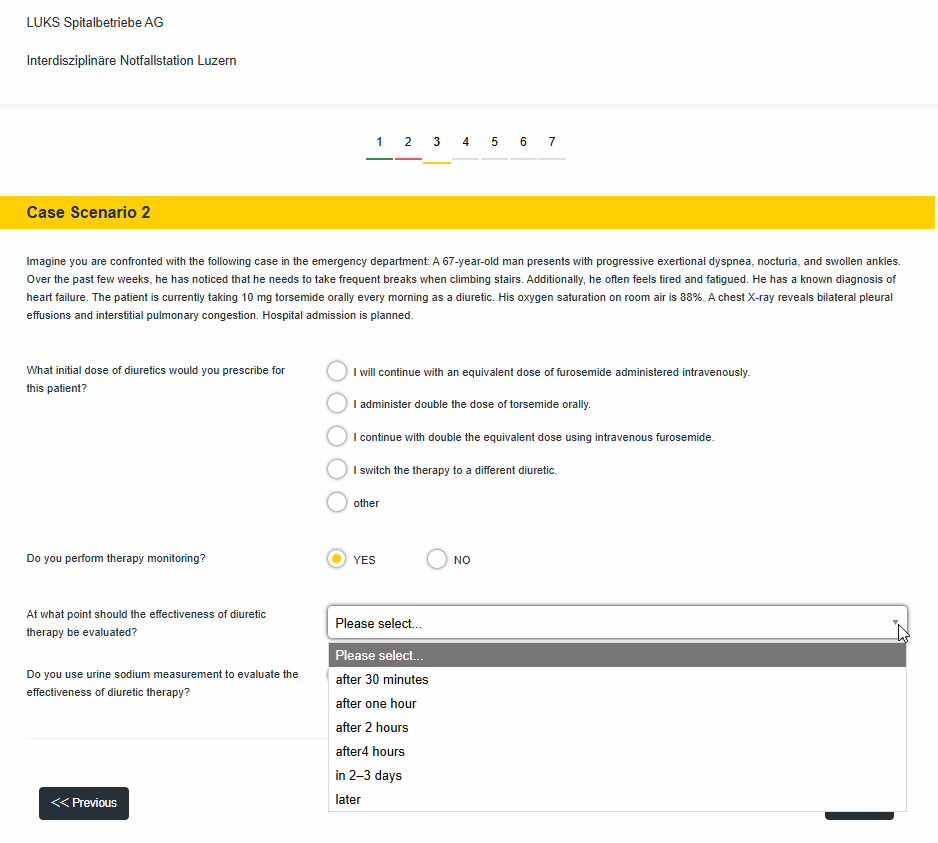


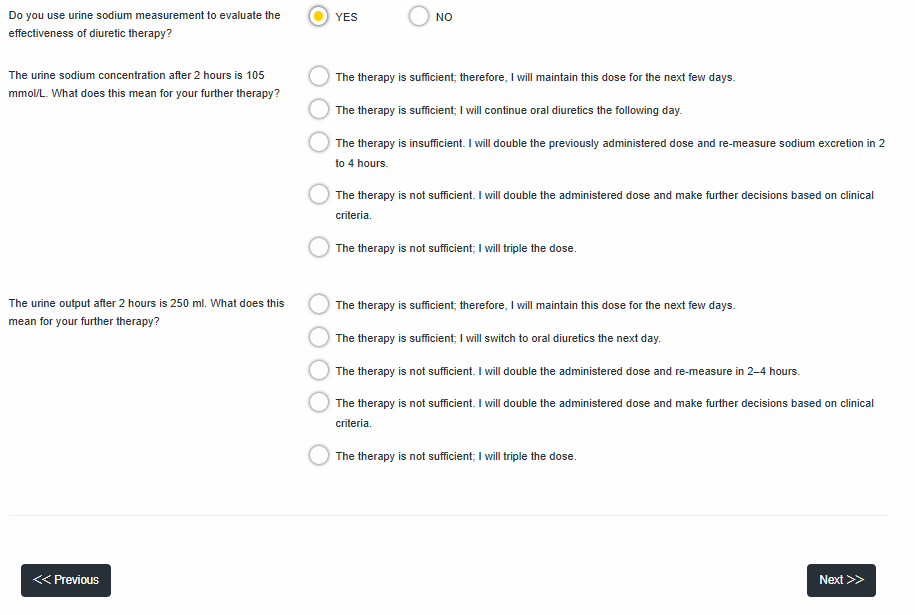


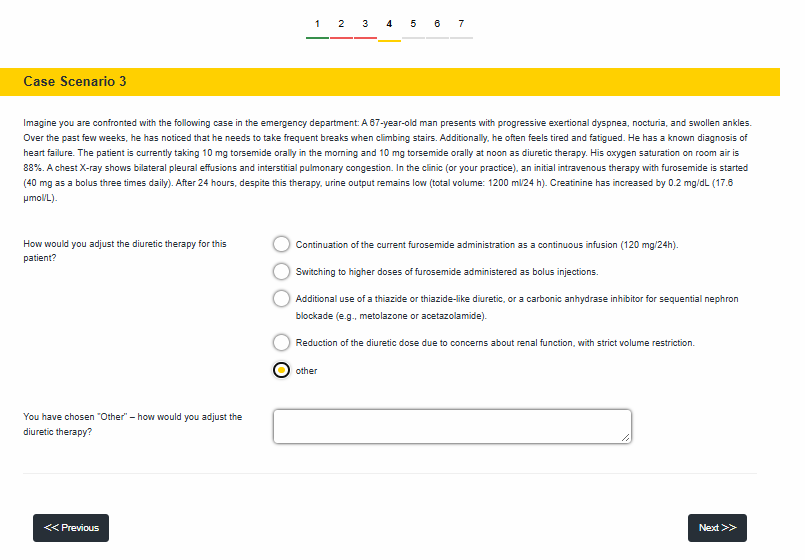


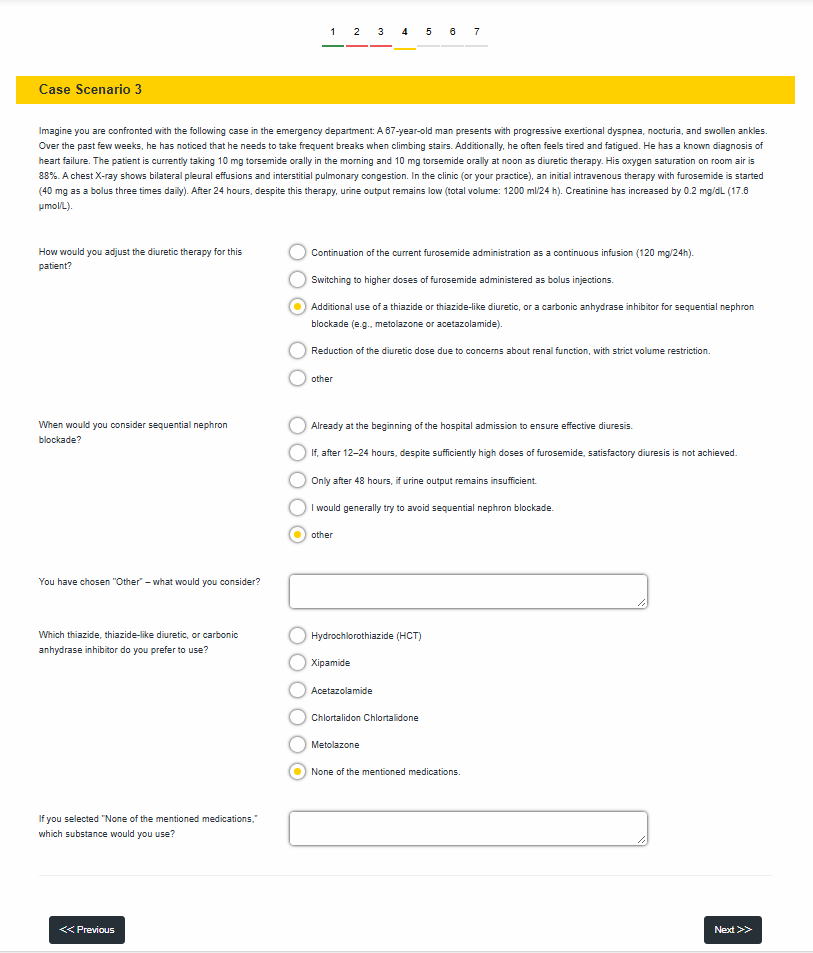


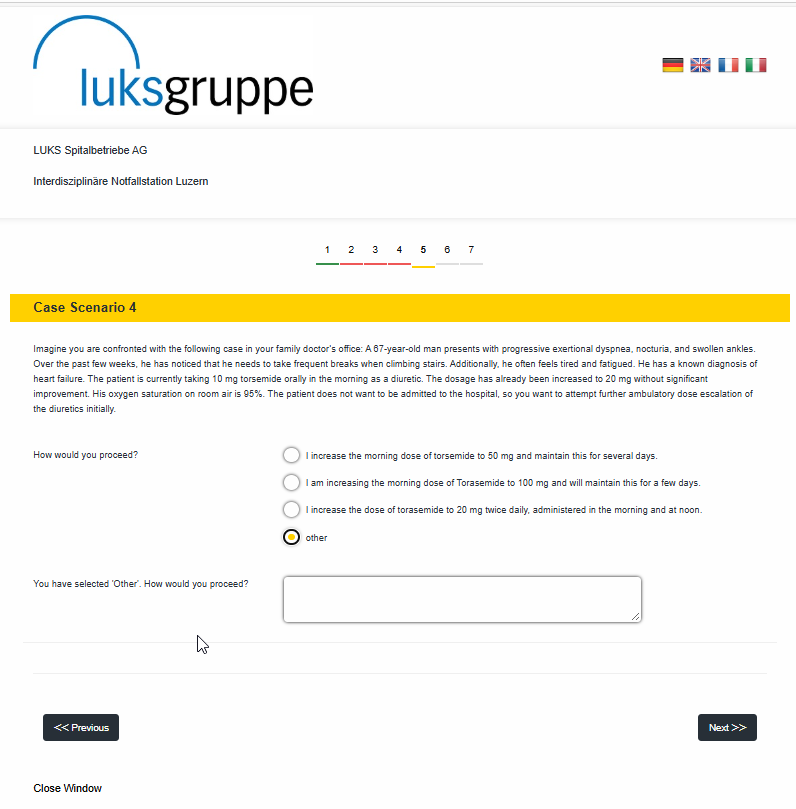


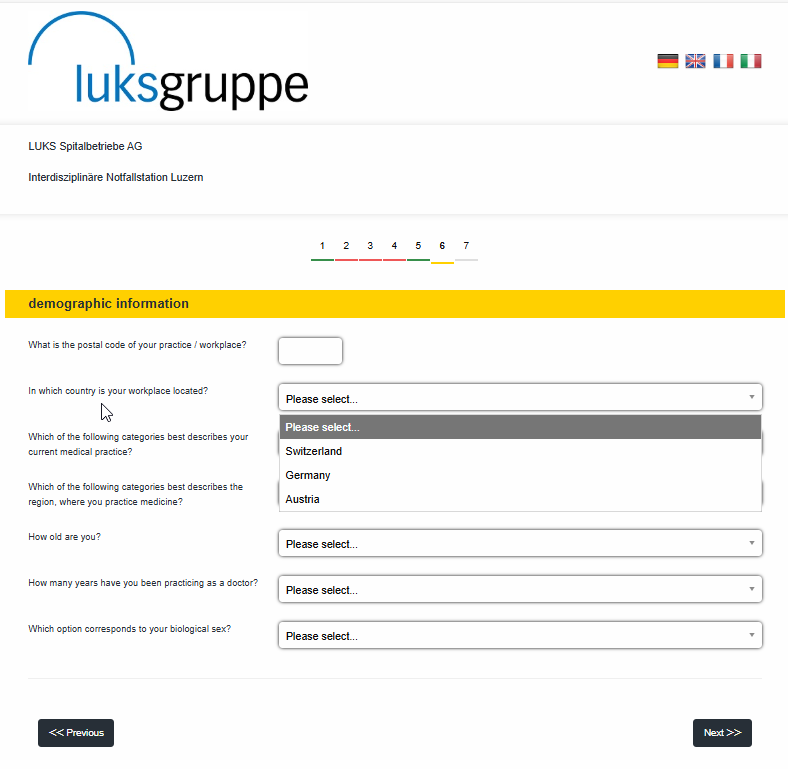


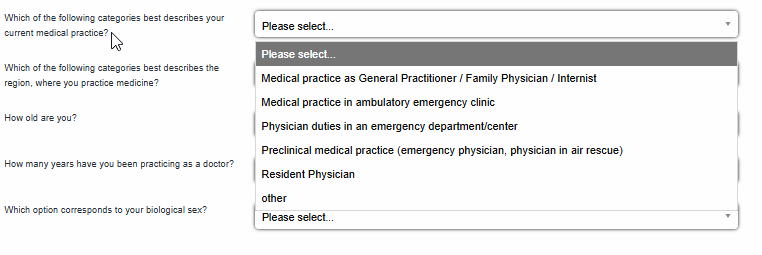


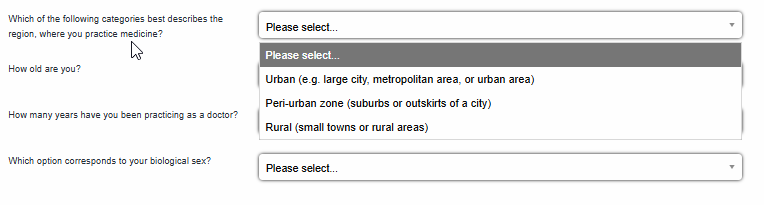


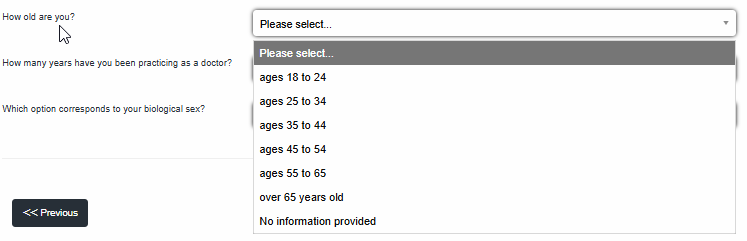


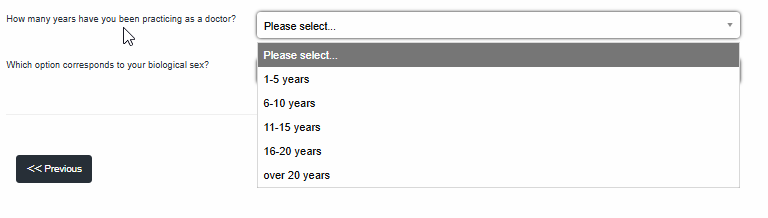


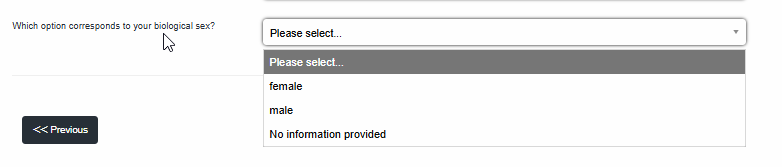


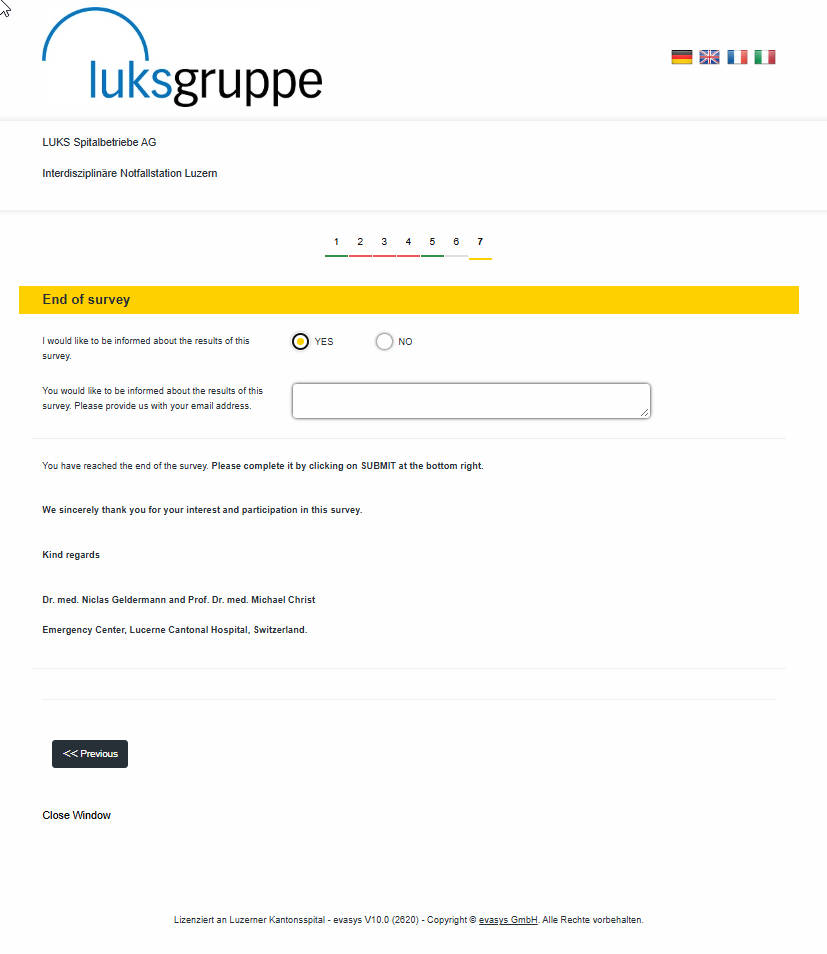

Supplement: xvag161_Supplementary_Data [file xvag161_supplementary_data.zip › Supplement 1.docx]
